# Impact of Implementing Evidence-Based Acute Stroke Interventions on Survival: The South London Stroke Register

**DOI:** 10.1371/journal.pone.0061581

**Published:** 2013-04-25

**Authors:** Juliet Addo, Siobhan Crichton, Ajay Bhalla, Anthony G. Rudd, Charles D. A. Wolfe, Christopher McKevitt

**Affiliations:** 1 Division of Health and Social Care Research, King’s College London, London, United Kingdom; 2 Department of Ageing and Health, Guy’s and St. Thomas’ NHS Foundation Trust, St Thomas’ Hospital, London, United Kingdom; 3 National Institute for Health Research Comprehensive Biomedical Research Centre, Guy’s and St. Thomas’ NHS Foundation Trust, London, United Kingdom; Cardiff University, United Kingdom

## Abstract

**Background:**

Studies examining the impact of organised acute stroke care interventions on survival in subgroups of stroke patients remain limited.

**Aims:**

This study examined the effects of a range of evidence-based interventions of acute stroke care on one year survival post-stroke and determined the size of the effect across different socio-demographic and clinical subgroups of patients.

**Methods:**

Data on 4026 patients with a first-ever stroke recruited to the population-based South London Stroke Register between 1995 and 2010 were used. In uni-variable analyses, one year cumulative survival rates in socio-demographic groups and by care received was determined. Survival functions were compared using Log-rank tests. Multivariable Cox models were used to test for interactions between components of care and age group, sex, ethnic group, social class, stroke subtype and level of consciousness.

**Results:**

1949 (56.4%) patients were admitted to a stroke unit. Patients managed on a stroke unit, those with deficits receiving specific rehabilitation therapies and those with ischaemic stroke subtype receiving aspirin in the acute phase had better one year survival compared to those who did not receive these interventions. The greatest reduction in the hazards of death among patients treated on a stroke unit were in the youngest patients aged <65 years, (HR 0.39; 95% CI: 0.25–0.62), and those with reduced levels of consciousness, GCS <9, (HR: 0.44; CI: 0.33–0.58).

**Conclusions:**

There was evidence of better one year survival in patients receiving specific acute interventions after stroke with a significantly greater effect in stroke subgroups, suggesting the possibility of re-organising stroke services to ensure that the most appropriate care is made accessible to patients likely to derive the most benefits from such interventions.

## Introduction

Organised inpatient care (stroke unit care), has been shown to improve outcome significantly after an acute stroke in randomised trials and observational studies in routine clinical practice.[1]–[4] Few studies, mostly trials, have examined longer term outcome (more than three months) with organised care after acute stroke and these have generally demonstrated long lasting benefits of stroke unit care up to 10 years after stroke.[5]–[9] Observational studies examining longer term outcome (≥3months) after stroke unit care have used national data from hospital registers, or in one study, compared two community populations where one had a stroke unit and the other did not. [5], [9] The survival advantage associated with stroke unit care has been shown to be relatively marked in specific subgroups of patients including younger patients, those with intracerebral haemorrhage and unconscious patients. [10] Previous studies from South London have demonstrated inequalities in access to organised inpatient care across different socio-demographic and clinical groups, but the impact of these on longer term outcome in subgroups of patients remain unknown. [11], [12] Availability of such data is necessary for reviewing opportunities for optimising access to stroke services and delivering longer term support for stroke patients, in line with the Department of Health of England’s, National Stroke Strategy. [13].

The aim of this study is to examine the associations between a range of evidence-based indicators of acute stroke care and death up to one year after a first-ever stroke in an unbiased population sample using data from the South London Stroke Register, and to determine the size of the effect across different socio-demographic and clinical subgroups of patients.

## Materials and Methods

### Ethics

Patients or their relatives gave written informed consent to participate in the study. The design of the study was approved by the ethics committees of Guy's and St Thomas' Hospital Trust, King's College Hospital, Queen’s Square, and Westminster Hospital (London).

### Identification of Stroke Patients

Patients were recruited from the South London Stroke Register (SLSR), an ongoing population-based study of incidence and outcome, that has prospectively recorded first-ever stroke in a geographically defined area of South London since 1995. At the 2001 Census, the population of the SLSR area was 271 817, with 63% whites; 9% black Caribbean; 15% black African and 13% other ethnic groups. The detailed methods of notification of patients and data collection have been described previously. [14], [15] In brief, patients were identified by register nurses and doctors using multiple sources of notification and recruited to the register as soon as possible after stroke onset. Data from patients who had their stroke between January 1995 and December 2010 were included in this study. Patients with subarachnoid haemorrhage were excluded from the analyses (n = 212) because the majority are managed in neurosurgical units following different protocols.

### Baseline Assessment

Data on demographic details include age; sex; self-defined ethnicity (white, black and other ethnicity) and socioeconomic status (Registrar General’s occupational codes grouped as manual and non-manual). Stroke severity measures obtained at the time of maximal impairment included urinary incontinence, Glasgow Coma Scale (GCS) classified as GCS <9, 9–12 and GCS ≥13, swallowing deficits (assessed by use of the 3-oz water swallow test) [16], stroke subtype (ischemic stroke, primary intracerebral haemorrhage and undefined), and pre-stroke Barthel Index (BI) for activities of daily living categorised as: 0 to 9 (severe disability), 10–14 (moderate disability) 15 to 19 (mild disability) and 20 (functionally independent). [17].

### Patterns of Care and Acute Interventions

Data were available on a range of evidence-based process indicators suggested to be used to assess the quality of acute stroke care.[18]–[20] These included admission to a stroke unit and spending more than 50% of hospital admission on a stroke unit. A stroke unit was defined as a discrete area of a hospital ward that took care of stroke patients and had a specialist multidisciplinary team providing a complex package of care to stroke patients in hospital. [2] Indicators of rehabilitation therapy provision (physiotherapy [PT] assessment within 72 hours; occupational therapy [OT] within seven days; and speech and language therapy [SALT]) within seven days for those with recorded deficits were examined for the period between 2005 and 2010 when the register collected data on these processes of care. The use of PT and OT was considered appropriate for patients who had any paralysis, visual field defects and sensory impairments in the acute phase. SALT was considered appropriate in patients with dysphasia, dysarthria, and dysphagia or failed swallow test. Another intervention for which data was collected between 2005 and 2010 only was the receipt of aspirin at anytime within the first week of stroke or within 48 hours if ischemic stroke.

### Outcome Measure

The outcome measure evaluated was the time of death in the first year after stroke. Patients with no record of death were censored at the end of 2011. Information on death was obtained from medical records, general practitioners and next-of kin and confirmed by the Office of National Statistics.

### Statistical Analysis

In univariable analyses, one year cumulative survival rates in socio-demographic groups and by care received were calculated, with 95% confidence intervals using Kaplan-Meier methods. Survival functions were compared using Log-rank tests. Multivariable Cox models were used to test for interactions between components of care and age group, sex, ethnic group, socioeconomic status, stroke subtype and level of consciousness. Models were also fitted within each group to compare the relative survival associated with receipt of care across different groups. All models were adjusted for age, gender, ethnicity, socioeconomic status, stroke subtype, GCS, incontinence and motor deficits. The year of stroke was included in all models to allow for long term time trends in care received on stroke units. [11] Receipt of aspirin within 48 hours was excluded from the multivariable analyses because of the low number of eligible patients not receiving it (n = 72 in total - and smaller in the subgroups). Patients with unknown and undefined stroke subtypes were excluded from multivariable analyses.

As interactions between patient group and care received were tested for 11 different interventions, a bonferroni adjustment was used and p<0.05/11 = 0.005 considered statistically significant in multivariate analyses. All analyses were conducted using STATA 11MP.

## Results

A total of 4026 patients with a first-ever stroke were registered between January 1995 and December 2010 with a mean age of 71.1 (SD 14.3) years. Table 1 shows the general characteristics and the cumulative survival by sub-groups of patients. Overall, 3535 (87.8%) of patients were admitted to hospital after their stroke and 1949 (56.4%) managed on a stroke unit. Between 2005 and 2010, 883 (84.1%) of patients with deficits received PT/OT and 550 (67.3%) received SALT. Patients who were managed on a stroke unit, those with deficits receiving specific rehabilitation therapies and those with ischaemic stroke who received aspirin in the acute phase had better one year survival compared to those who did not receive such acute care interventions.

**Table 1:** General characteristics and cumulative survival at one year post stroke.

	N (%)	Cumulative survival (95% CI)	p-value		N (%)	Cumulativesurvival (95% CI)	p-value
All	4026			**Care indicators**			
**Gender**			<0.0001	**Hospital** **admission**			<0.0001
Male	2033(50.5)	73.9(71.9–79.0)		Yes	3535(87.8)	65.3(63.6–66.8)	
Female	1993(49.5)	62.3(60.1–64.4)		No	491(12.2)	90.9(87.8–93.2)	
**Age (years)**			<0.0001	**Stroke unit** **admission**			<0.0001
<65	1172(29.1)	85.0(82.9–87.0)		Yes	1949(56.4)	72.9(70.9–74.8)	
65–74	1050(26.1)	74.7(71.9–77.2)		No	1510(43.7)	54.6(52.1–57.2)	
75–84	1175(29.2)	60.0(57.1–62.8)		**50% of stay on** **a stroke unit**			<0.0001
85+	629(15.6)	40.4(36.5–44.3)		Yes	1451(46.5)	74.5(72.2–76.7)	
**Ethnicity**			<0.0001	No	1667(53.5)	55.9(53.5–58.3)	
White	2894(73.7)	64.3(62.5–66.0)		**Brain imaging**			<0.0001
Black	806(20.5)	80.1(77.1–82.7)		Yes	3595(93.0)	70.3(68.8–71.8)	
Other	228(5.8)	81.3(75.5–85.8)		No	269(7.0)	29.9(23.8–36.1)	
**Socioeconomic status**			0.0108	**Swallow test done**			0.0680
Non-manual	1056(33.0)	80.6(78.0–82.9)		Yes	3216(91.6)	65.6(63.9–67.2)	
Manual	2141(67.0)	72.6(70.7–74.5)		No	295(8.4)	61.7(55.8–67.1)	
**Subtype**			<0.0001	**Aspirin given in** **the acute phase**			<0.0001
Infarction	3149(78.2)	71.4(69.8–77.5)		Yes	840(85.0)	78.1(75.1–80.7)	
PICH	544(13.5)	57.3(52.8–61.5)		No	148(15.0)	59.6(51.2–67.0)	
Undefined	187(4.6)	28.9(22.2–35.9)		**Aspirin given** **within 48 hrs**			0.1369
Unknown	146(3.6)	80.9(73.5–86.5)		Yes	629(89.7)	77.4(73.9–80.5)	
**GCS**			<0.0001	No	72(10.3)	84.7(79.0–94.3)	
<9	584(15.2)	18.7(15.4–22.2)		**PTOT**			<0.0001
9–15	470(12.2)	48.8(44.2–53.3)		Yes	883(84.1)	73.8(70.7–76.6)	
13–15	2802(72.7)	80.3(78.8–81.8)		No	167(15.9)	51.9(43.8–59.4)	
				**SALT**			<0.0001
				Yes	550(67.3)	70.4(66.4–74.0)	
				No	267(32.7)	56.8(50.5–62.6)	

Multivariate analyses testing for interactions between components of acute stroke care and socio-demographic as well as clinical sub-groups of patients are shown in Tables 2–5. There was a significant interaction between age and stroke unit care as shown in Table 2 (p = 0.0018). The relative effect was greatest in younger patients (<65 year olds) where stroke unit admission was associated with a 61% decrease in hazard of death (HR 0.39; 95% CI: 0.25–0.62) compared to a 22% reduction in hazard (HR 0.78; 95% CI: 0.57–1.05) in older patients (85+ years). There was however no significant interaction between age and the other acute care interventions examined.

**Table 2:** Relative hazard of death following receipt of acute care interventions by age group.

	Age, HR(95%CI)				
	<65 years	65–74 years	75–84 years	85+ years	p-value**
Hospital admission	2.38(0.74–7.68)	2.30(1.06–4.98)	2.531.32–4.84)	4.06(1.65–9.97)	0.9980
Stroke unit admission	0.39(0.25–0.62)	0.64(0.46–0.91)	0.66(0.51–0.86)	0.78(0.57–1.05)	0.0018
50% of stay on a stroke unit	0.44(0.25–0.75)	0.89 0.61–1.28)	0.71(0.83–0.95)	0.74(0.53–1.05)	0.0038
50% of stay on a stroke unit*	1.07(0.42–2.76)	1.22(0.69–2.17)	1.15(0.73–1.82)	0.85(0.51–1.42)	0.5279
Brian imaging	0.18(0.05–0.57)	0.62(0.22–1.77)	0.33(0.19–0.56)	0.54(0.30–0.97)	0.4115
Swallow test done	0.59(0.35–0.99)	0.74(0.44–1.25)	0.83(0.47–1.47)	0.84(0.40–1.46)	0.3912
Aspirin given in the acute phase	0.34(0.12–0.97)	0.47(0.21–0.94)	0.42(0.23–0.74)	0.41(0.19–0.85)	0.3699
PTOT	0.22(0.08–0.62)	0.25(0.13–0.48)	0.31(0.17–0.57)	0.39(0.20–0.74)	0.2432
PTOT*	0.32(0.08–1.26)	0.22(0.11–0.46)	0.25(0.12–0.52)	0.15(0.06–0.39)	0.8349
SALT	0.30(0.11–0.84)	0.40(0.22–0.74)	0.32(0.18–0.56)	0.41(0.22–0.78)	0.2374
SALT*	0.45(0.15–1.38)	0.44(0.21–0.92)	0.46(0.23–0.93)	0.36(0.16–0.83)	0.9979

* excluding patients not admitted to a su.

** P<0.005 considered statistically significant.

**Table 3:** Relative hazard of death following receipt of acute care interventions by gender.

	Gender, HR(95%CI)		
	Male	Female	p-value**
Hospital admission	2.11(1.20–3.72)	3.39(1.89–6.08)	0.7927
Stroke unit admission	0.59(0.46–0.75)	0.67(0.54–0.82)	0.2305
50% of stay on a stroke unit	0.68(0.52–0.90)	0.75(0.60–0.94)	0.2793
50% of stay on a stroke unit*	0.95(0.62–1.43)	1.14(0.79–1.66)	0.4317
Brian imaging	0.33(0.19–0.60)	0.48(0.31–0.74)	0.2146
Swallow test done	0.72(0.49–1.06)	0.78(0.55–1.11)	0.3562
Aspirin given inthe acute phase	0.36(0.21–0.61)	0.53(0.32–0.86)	0.5006
PTOT	0.24(0.14–0.39)	0.38(0.24–0.59)	0.4347
PTOT*	0.29(0.14–0.57)	0.40(0.21–0.77)	0.3930
SALT	0.52(0.33–0.91)	0.30(0.20–0.45)	0.0987
SALT*	0.85(0.48–1.50)	0.26(0.15–0.45)	0.0101

* excluding patients not admitted to a su.

** P<0.005 considered statistically significant.

**Table 4:** Relative hazard of death following receipt of acute care interventions by socioeconomic status and ethnicity.

	Socioeconomic status, HR(95%CI)			Ethnicity, HR(95%CI)		
	Non-Manual	Manual	p-value**	White	Black	p-value**
Hospital admission	2.71(1.17–6.25)	2.29(1.39–3.78)	0.5697	3.20(2.01–5.09)	1.54(0.55–4.31)	0.2593
Stroke unit admission	0.54(0.39–0.76)	0.74(0.59–0.94)	0.4719	0.64(0.54–0.76)	0.62(0.42–0.92)	0.4358
50% of stay on a stroke unit	0.60(0.41–0.87)	0.69(0.53–0.89)	0.7237	0.70(0.57–0.84)	0.82(0.53–1.27)	0.5932
50% of stay on a stroke unit*	0.84(0.42–1.69)	0.81(0.57–1.16)	0.3446	0.96(0.71–1.29)	1.21(0.62–2.37)	0.4759
Brian imaging	0.33(0.10–1.08)	0.64(0.36–1.12)	0.4287	0.44(0.31–0.64)	0.47(0.15–1.45)	0.4022
Swallow test done	0.47(0.26–0.83)	0.69(0.47–1.02)	0.1549	0.64(0.48–0.84)	1.24(0.58–2.65)	0.2254
Aspirin given inthe acute phase	0.37(0.15–0.90)	0.57(0.31–1.03)	0.4588	0.47(0.32–0.70)	0.24(0.09–0.62)	0.6308
PTOT	0.56(0.47–1.17)	0.25(0.14–0.44)	0.0648	0.34(0.24–0.50)	0.31(0.14–0.69)	0.6803
PTOT*	0.18(0.04–0.79)	0.33(0.15–0.71)	0.4278	0.32(0.19–0.54)	0.35(0.11–1.10)	0.6803
SALT	0.45(0.20–0.99)	0.63(0.38–1.06)	0.7169	0.39(0.27–0.56)	0.34(0.16–0.73)	0.6352
SALT*	0.59(0.18–1.90)	0.60(0.32–1.13)	0.3341	0.47(0.30–0.75)	0.49(0.19–1.13)	0.7488

* excluding patients not admitted to a su.

** P<0.005 considered statistically significant.

**Table 5:** Relative hazard of death following the receipt of acute care interventions by stroke subtype and GCS.

	Subtype, HR (95% CI)			GCS, HR (95% CI)			
	Infarction	PICH	p-value**	<9	9–12	13–15	p-value**
Stroke unit admission	0.74(0.62–0.89)	0.39(0.27–0.57)	0.0052	0.44(0.33–0.58)	0.48(0.33–0.70)	0.98(0.78–1.23)	0.0238
50% of stay on a stroke unit	0.79(0.66–0.96)	0.56(0.37–0.86)	0.0875	0.60(0.45–0.81)	0.73(0.49–1.07)	0.93(0.72–1.22)	0.8396
50% of stay on a stroke unit*	1.05(0.78–1.41)	1.35(0.68–2.68)	0.8410	1.27(0.75–2.17)	1.52(0.85–2.69)	0.77(0.51–1.15)	0.3486
Brian imaging	0.43(0.30–0.62)	0.47(0.14–1.58)	0.2520	0.37(0.23–0.62)	0.49(0.19–1.30)	0.90(0.48–1.66)	0.2929
Swallow test done	0.78(0.57–1.07)	0.68(0.41–1.12)	0.1014	0.66(0.45–0.96)	0.38(0.21–0.71)	1.17(0.73–1.87)	0.0134
Aspirin given inthe acute phase	NA	NA	NA	0.33(0.18–0.61)	0.29(0.12–0.67)	0.77(0.43–1.36)	0.0007
PTOT	0.47(0.32–0.68)	0.07(0.03–0.22)	<0.0001	0.13(0.07–0.24)	0.20(0.09–0.46)	0.83(0.40–1.71)	<0.001
PTOT*	0.46(0.27–0.80)	0.04(0.01–0.22)	0.0041	0.10(0.04–0.23)	0.16(0.04–0.73)	0.85(0.36–2.01)	0.0005
SALT	0.45(0.32–0.64)	0.21(0.09–0.45)	0.0141	0.15(0.09–0.26)	0.12(0.05–0.29)	1.38(0.76–2.48)	<0.001
SALT*	0.52(0.34–0.81)	0.26(0.09–0.76)	0.2736	0.11(0.05–0.25)	0.21(0.05–0.95)	1.32(0.70–2.48)	<0.001

* excluding patients not admitted to a su.

** P<0.005 considered statistically significant.

NA not applicable.

After allowing for multiple testing, there were no statistically significant interactions between the receipt of careand gender (Table 3), ethnicity or socioeconomic status (Table 4).

Table 5 shows the interactions between acute care interventions and clinical sub groups of patients. Although not statistically significant and the p<0.005 level,s, there was a borderline interaction (p = 0.0052) between stroke unit care and subtype with stroke unit admission associated with a 61% reduction in the hazard of death in patients with a haemorrhagic stroke (HR: 0.39; CI: 0.27–0.57) compared to 26% reduction in those with ischaemic stroke (HR: 0.74; 95% CI: 0.62–0.89). There were significant interactions between stroke subtype and the receipt of PT/OT. and between the level of consciousness (GCS level) and the receipt of aspirin and rehabilitation therapy in the acute phase. The largest reductions in the hazards of death in these instances were observed in unconscious patients (GCS <9) compared to those with higher levels of consciousness (GCS 13–15).

## Discussion

This population-based study showed a better one year survival in patients who received acute care interventions including stroke unit care, rehabilitation therapies and aspirin after an acute stroke compared to those who did not. The largest reduction in the hazards of death among patients managed on a stroke unit were observed in younger patients (<65 years) compared to older patients, those with a haemorrhagic stroke compared to an infarct, and patients who were unconscious at the time of admission (GCS <9) compared to conscious patients (GCS ≥9). Receipt of aspirin among patients with ischaemic stroke and rehabilitation therapy where there were deficits, were associated with better one year survival in unconscious patients (GCS <9) compared to more conscious patients (GCS ≥9). These findings were independent of stroke unit care.

The findings of better survival after receipt of acute care interventions in this study are similar to previously reported results from randomised trials.[21]–[23] Few studies have examined the effect of acute care interventions on subgroups of stroke patients in observational studies.[24]–[26] With the exception of patients with intracranial haemorrhage who had better outcome when managed on a stroke unit in an observational study which included stroke patients admitted to several hospitals in Italy, there were no significant interactions between stroke unit care and other patient characteristics. [26] Similar to the findings of our study however, results from the Riks-Stroke study involving all hospitals admitting stroke patients in Sweden, demonstrated better survival associated with stroke unit care in all patients, with greater effect in younger patients, those with intracerebral haemorrhage and patients who were unconscious. [25] Significant differences in management and complications between stroke units and general wards have been reported as possible explanations for the more favourable outcome seen in patients on stroke units compared to general wards. [27], [28] Patients with more severe strokes may require more specialised care compared to milder strokes and such care is probably best provided in a stroke unit than on a general ward. This may be a possible explanation for the survival advantage observed in such patients with stroke unit care. These findings may be relevant in situations where difficulties in accessing acute care interventions such as unavailability of stroke unit beds may warrant prioritisation of stroke patients according to those likely to have the greatest benefits. It is however possible that these findings could have been different for patients with milder strokes if the outcome measure was dependency and not death as used in this study. Interestingly, just receiving care on a stroke unit and not necessarily the proportion of hospital admission time spent receiving such an intervention, had beneficial effects on one year survival. Although, these findings were adjusted for case mix differences this may be due to unmeasured confounders. If patients with the poorest prognosis are those spending the longest time on a stroke unit, this could potentially confound results and mask any survival advantages associated with spending a greater proportion of hospital stay on a stroke unit This study also showed the beneficial effects of acute care interventions beyond stroke unit care, such as rehabilitation therapies and aspirin therapy in patients with reduced levels of consciousness, emphasising the need to ensure accessibility of appropriate interventions to patients regardless of stroke severity and subtype.

A previous study from this multiethnic South London population reported inequalities in access to acute care interventions with patients of black ethnicity more likely to be admitted to a stroke unit, and older patients as well as those of lower socioeconomic status less likely to have brain imaging. [11] The results of the present study provide no evidence to justify the existence of such inequalities to acute care interventions, as there was no survival advantage observed in these subgroups of patients above others.

This study controlled for case mix differences in the analyses, but there could still be potential for residual confounding. The inequalities described above, in access to some of the interventions may potentially lead to bias when determining the size of the association between intervention and survival. However, unless different, unmeasured, selection criteria were being applied to patients in each subgroup assessed in this study, it is unlikely unmeasured confounders would bias the interaction between intervention and subgroup.

The various components of stroke unit care considered were by no means exhaustive, and additionally, although the organised care components included rehabilitation therapies, it did not consider the number of therapy sessions and the duration between onset of symptoms and receipt of these therapies which could potentially affect outcome. In the future assessing whether intensity of therapies and contact with the wider multidisciplinary team has increased beneficial impact on outcome within certain subgroups of patients would be of interest. It is also possible that some patients with severe clinical impairments and co- morbidities or those who developed complications after stroke were admitted, or transferred, to other wards apart from stroke units, which could potentially have influenced the outcome examined in the study.

Despite these limitations, the present study using detailed information from a population-based stroke register with high case-ascertainment rates (shown to be around 80%) and with unselected patients has enabled us to examine the effects of implementing evidence-based acute stroke care interventions on one year survival. [29] The present study using detailed information from a population-based stroke register with high case-ascertainment rates (shown to be around 80%) and with unselected patients has enabled us to examine the effects of implementing evidence-based acute stroke care interventions on one year survival. [29] A strength of this study is the ability to study the effect of the acute care interventions on subgroups of stroke patients. As a result of the cohort design of the study, information on the patients’ characteristics and interventions received were collected before the onset of the outcome (death) by independent observers who were unaware of any study hypothesis, thus limiting the possibility of bias occurring. Information on death was also complete and was confirmed by the UK Office of National Statistics.

In conclusion, the findings of this study suggest an improvement in one year survival in patients receiving specific acute care interventions after stroke with a significantly greater effect observed in younger patients, those with haemorrhagic strokes and those with reduced level of consciousness in the acute phase in this multiethnic population. These findings provide a platform upon which to re-organise the delivery of acute stroke care to encourage universal access to these services, with the possibility of ensuring that subgroups of patients shown to derive the most benefits from acute care interventions receive the most appropriate care as a matter of priority.

## References

[1] Govan L, Weir CJ, Langhorne P, for the Stroke Unit Trialists C (2008) Organized Inpatient (Stroke Unit) Care for Stroke. Stroke 39: 2402–2403.

[2] Stroke Unit Trialists C (2007) Organised inpatient (stroke unit) care for stroke. Cochrane Database Syst Rev DOI: 10.1002/14651858.CD000197.pub2. 10.1002/14651858.CD000197.pub2 17943737

[3] Seenan P, Long M, Langhorne P (2007) Stroke units in their natural habitat: systematic review of observational studies. Stroke 38: 1886–1892. 17463308 10.1161/STROKEAHA.106.480871

[4] Langhorne P, Lewsey JD, Jhund PS, Gillies M, Chalmers JW, et al (2010) Estimating the impact of stroke unit care in a whole population: an epidemiological study using routine data. J Neurol Neurosurg Psychiatry 81: 1301–1305. 20601665 10.1136/jnnp.2009.195131

[5] Jorgensen HS, Kammersgaard LP, Nakayama H, Raaschou HO, Larsen K, et al (1999) Treatment and rehabilitation on a stroke unit improves 5-year survival. A community-based study. Stroke 30: 930–933. 10229722 10.1161/01.str.30.5.930

[6] Indredavik B, Bakke F, Slordahl SA, Rokseth R, Haheim LL (1999) Stroke unit treatment. 10-year follow-up. Stroke 30: 1524–1527. 10436094 10.1161/01.str.30.8.1524

[7] Indredavik B, Slordahl SA, Bakke F, Rokseth R, Haheim LL (1997) Stroke unit treatment. Long-term effects. Stroke 28: 1861–1866. 9341685 10.1161/01.str.28.10.1861

[8] Drummond AE, Pearson B, Lincoln NB, Berman P (2005) Ten year follow-up of a randomised controlled trial of care in a stroke rehabilitation unit. Bmj 331: 491–492. 16093255 10.1136/bmj.38537.679479.E0 PMC1199027

[9] Glader EL, Stegmayr B, Johansson L, Hulter-Asberg K, Wester PO (2001) Differences in long-term outcome between patients treated in stroke units and in general wards: a 2-year follow-up of stroke patients in sweden. Stroke 32: 2124–2130. 11546906 10.1161/hs0901.095724

[10] Terent A, Asplund K, Farahmand B, Henriksson KM, Norrving B, et al (2009) Stroke unit care revisited: who benefits the most? A cohort study of 105,043 patients in Riks-Stroke, the Swedish Stroke Register. J Neurol Neurosurg Psychiatry 80: 881–887. 19332423 10.1136/jnnp.2008.169102

[11] Addo J, Bhalla A, Crichton S, Rudd AG, McKevitt C, et al.. (2011) Provision of acute stroke care and associated factors in a multiethnic population: prospective study with the South London Stroke Register. Bmj DOI: 10.1136/bmj.d744. 10.1136/bmj.d744 PMC3044771 21349892

[12] McKevitt C, Coshall C, Tilling K, Wolfe C (2005) Are there inequalities in the provision of stroke care? Analysis of an inner-city stroke register. Stroke 36: 315–320. 15618440 10.1161/01.STR.0000152332.32267.19

[13] Department of Health website. Available: http://www.dh.gov.uk/en/Publicationsandstatistics/Publications/PublicationsPolicyAndGuidance/DH_081062. Accessed 2012 Nov 21.

[14] Tilling K, Sterne JA, Wolfe CD (2001) Estimation of the incidence of stroke using a capture-recapture model including covariates. Int J Epidemiol 30: 1351–1359. 11821345 10.1093/ije/30.6.1351

[15] Stewart JA, Dundas R, Howard RS, Rudd AG, Wolfe CD (1999) Ethnic differences in incidence of stroke: prospective study with stroke register. Bmj 318: 967–971. 10195965 10.1136/bmj.318.7189.967 PMC27822

[16] DePippo KL, Holas MA, Reding MJ (1992) Validation of the 3-oz water swallow test for aspiration following stroke. Arch Neurol 49: 1259–1261. 1449405 10.1001/archneur.1992.00530360057018

[17] Wolfe CD, Taub NA, Woodrow EJ, Burney PG (1991) Assessment of scales of disability and handicap for stroke patients. Stroke 22: 1242–1244. 1833860 10.1161/01.str.22.10.1242

[18] Kjellstrom T, Norrving B, Shatchkute A (2007) Helsingborg Declaration 2006 on European stroke strategies. Cerebrovasc Dis 23: 231–241. 17139166 10.1159/000097646

[19] Royal College of Physicians London Bookshop. Available: http://bookshop.rcplondon.ac.uk/contents/6ad05aab-8400-494c-8cf4-9772d1d5301b.pdf. Accessed 2012 Nov 21.

[20] Adams HP Jr, del Zoppo G, Alberts MJ, Bhatt DL, Brass L, et al (2007) Guidelines for the early management of adults with ischemic stroke: a guideline from the American Heart Association/American Stroke Association Stroke Council, Clinical Cardiology Council, Cardiovascular Radiology and Intervention Council, and the Atherosclerotic Peripheral Vascular Disease and Quality of Care Outcomes in Research Interdisciplinary Working Groups: the American Academy of Neurology affirms the value of this guideline as an educational tool for neurologists. Stroke 38: 1655–1711. 17431204 10.1161/STROKEAHA.107.181486

[21] Indredavik B, Bakke F, Slordahl SA, Rokseth R, Haheim LL (1998) Stroke unit treatment improves long-term quality of life: a randomized controlled trial. Stroke 29: 895–899. 9596231 10.1161/01.str.29.5.895

[22] Kalra L, Evans A, Perez I, Knapp M, Swift C, et al.. (2005) A randomised controlled comparison of alternative strategies in stroke care. Health Technol Assess 9: iii–iv, 1–79. 10.3310/hta9180 15890138

[23] Ronning OM, Guldvog B (1998) Stroke units versus general medical wards, I: twelve- and eighteen-month survival: a randomized, controlled trial. Stroke 29: 58–62. 9445329 10.1161/01.str.29.1.58

[24] Stegmayr B, Asplund K, Hulter-Asberg K, Norrving B, Peltonen M, et al (1999) Stroke units in their natural habitat: can results of randomized trials be reproduced in routine clinical practice? Riks-Stroke Collaboration. Stroke 30: 709–714. 10187866 10.1161/01.str.30.4.709

[25] Terent A, Asplund K, Farahmand B, Henriksson KM, Norrving B, et al (2009) Stroke unit care revisited: who benefits the most? A cohort study of 105,043 patients in Riks-Stroke, the Swedish Stroke Register. J Neurol Neurosurg Psychiatry 80: 881–887. 19332423 10.1136/jnnp.2008.169102

[26] Candelise L, Gattinoni M, Bersano A, Micieli G, Sterzi R, et al (2007) Stroke-unit care for acute stroke patients: an observational follow-up study. Lancet 369: 299–305. 17258670 10.1016/S0140-6736(07)60152-4

[27] Indredavik B, Bakke F, Slordahl SA, Rokseth R, Haheim LL (1999) Treatment in a combined acute and rehabilitation stroke unit: which aspects are most important? Stroke 30: 917–923. 10229720 10.1161/01.str.30.5.917

[28] Evans A, Perez I, Harraf F, Melbourn A, Steadman J, et al (2001) Can differences in management processes explain different outcomes between stroke unit and stroke-team care? Lancet 358: 1586–1592. 11716885 10.1016/S0140-6736(01)06652-1

[29] Heuschmann PU, Grieve AP, Toschke AM, Rudd AG, Wolfe CD (2008) Ethnic group disparities in 10-year trends in stroke incidence and vascular risk factors: the South London Stroke Register (SLSR). Stroke 39: 2204–2210. 18535279 10.1161/STROKEAHA.107.507285

